# A Rare Case of Bladder Melanosis: A Case Report and Review of the Literature

**DOI:** 10.7759/cureus.75604

**Published:** 2024-12-12

**Authors:** Ben Walters, Srinath Ileperuma, Andleeb Abrari, Mohammad Fani

**Affiliations:** 1 Urology, Rotherham District General Hospital, Rotherham, GBR; 2 Histopathology, Rotherham District General Hospital, Rotherham, GBR

**Keywords:** bladder melanosis, melanoma diagnosis, melanosis, review of the literature, transitional cell carcinoma (tcc)

## Abstract

Bladder melanosis is a rare and poorly understood condition involving melanin pigmentation within the urothelial mucosa. Cases often present with haematuria, urinary obstructive symptoms, or cystitis. While generally considered benign, its potential association with malignancy warrants regular monitoring, as cases have previously been reported of an association with urothelial carcinoma and melanoma, although it is unclear whether there is a causal relationship. Here, we present the case of a fit and well 51-year-old male who was diagnosed incidentally with bladder melanosis following cystoscopy and perform a thorough literature review of all known cases.

## Introduction

Melanosis is defined in medical dictionaries as a condition characterised by abnormal deposition of melanin, or sometimes other pigments, in the tissues of the body [1]. This does not imply that the pigment has to be melanin, and in fact, the black discolouration of colonic mucosa, termed melanosis coli, is a result of the accumulation of lipofuscin [2].

Melanosis most commonly occurs in the skin and mucocutaneous tissues such as the oral cavity, colon, genitalia, and conjunctiva [3]. Melanosis of the bladder was first described by Alroy et al. in 1986 and is a rare entity [4]. It is usually discovered incidentally when patients undergo cystoscopy to investigate non-specific urinary tract symptoms or haematuria. On cystoscopy, it is seen as a dark brown, velvety appearance that is patchy or multifocal [5]. It most frequently affects middle-aged and older adults aged between 43 and 86, but cases have been reported in those as young as five [6].

The underlying aetiology and significance are obscure, and a paucity of data exists on long-term follow-up and outcomes.

In this article, we describe a case of bladder melanosis in a fit and well 51-year-old who presented with visible haematuria on a background of long-term urinary frequency and nocturia. We also review all available literature and synthesise all current known cases.

## Case presentation

A fit and well 51-year-old male who had never smoked and drank 34 units of alcohol per week was referred by their general practitioner to our district general hospital following two episodes of visible haematuria on a background of long-term urinary frequency and nocturia.

**Figure 1:**
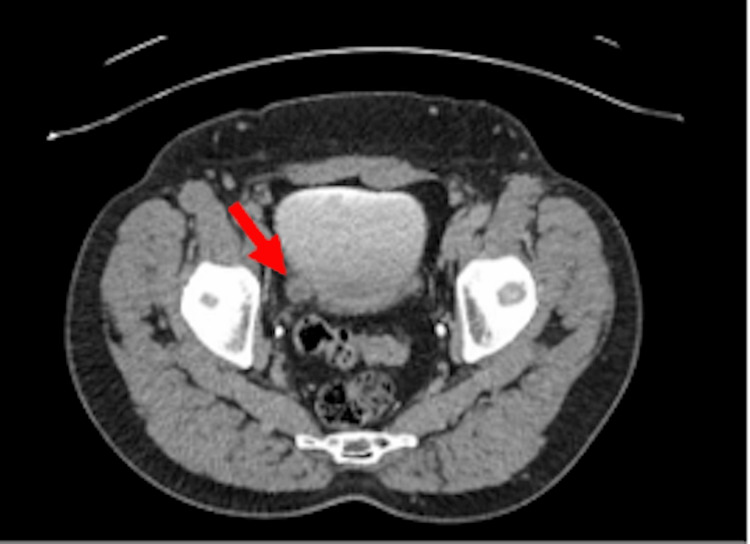
Computed tomography (CT) scan Post-contrast CT showing bladder diverticula (red arrow)

**Figure 2:**
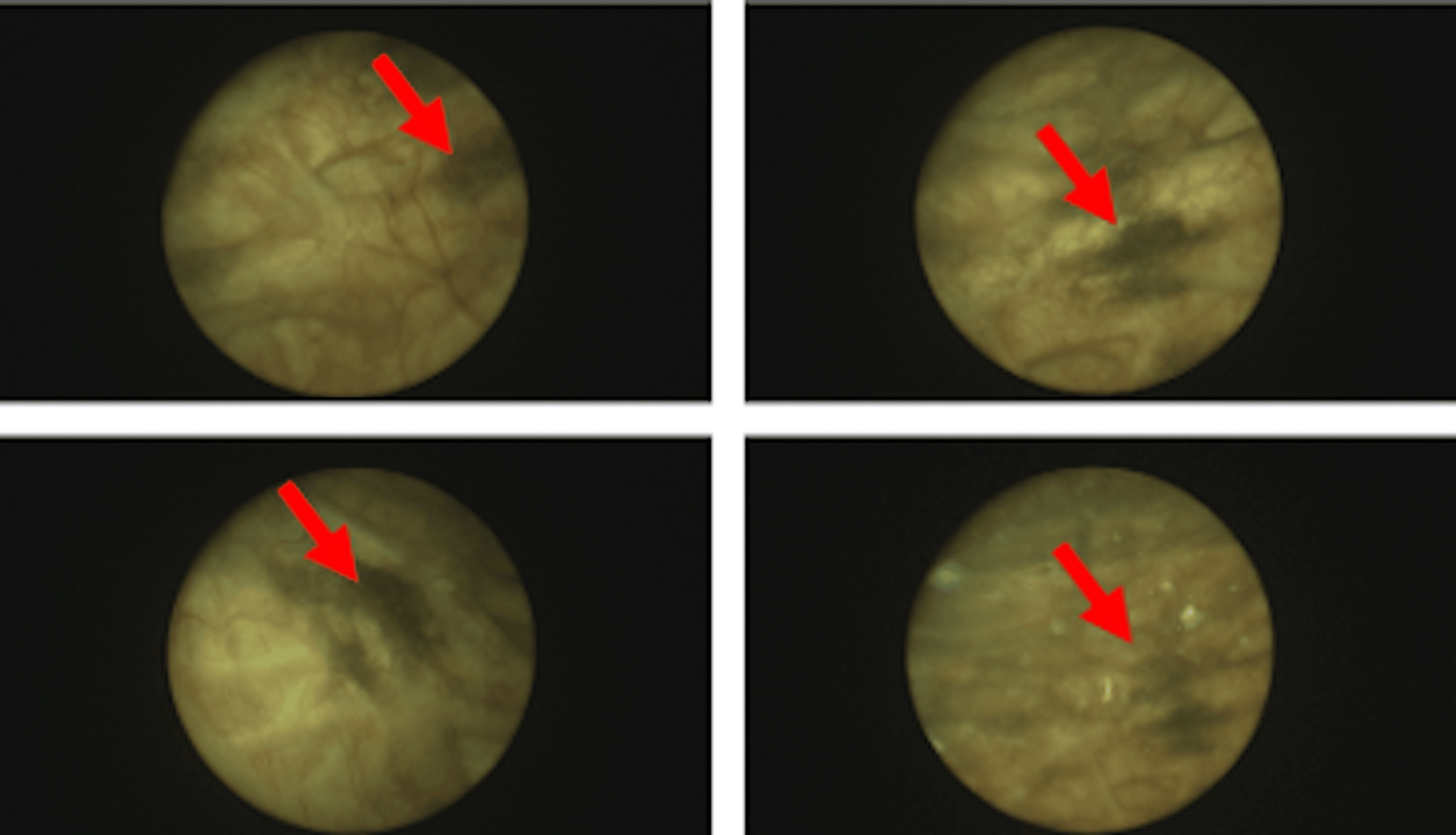
Cystoscopic appearances of the patient’s bladder Dark patches are seen on the bladder mucosa (red arrows).

The initial urine dip was positive for blood, leucocytes, and nitrites. Subsequent urine microscopy and blood tests were unremarkable. A computed tomography urogram with contrast showed multiple small diverticula on the right lateral wall of the urinary bladder (Figure 1), but it was otherwise normal. Uroflow studies showed a maximum flow rate of 22.9 ml per second and a residual urine volume of 317 ml. Cystoscopy revealed a highly trabeculated bladder with multiple diverticular and widespread black deposits (Figure 2) throughout the bladder, which were biopsied.

**Figure 3:**
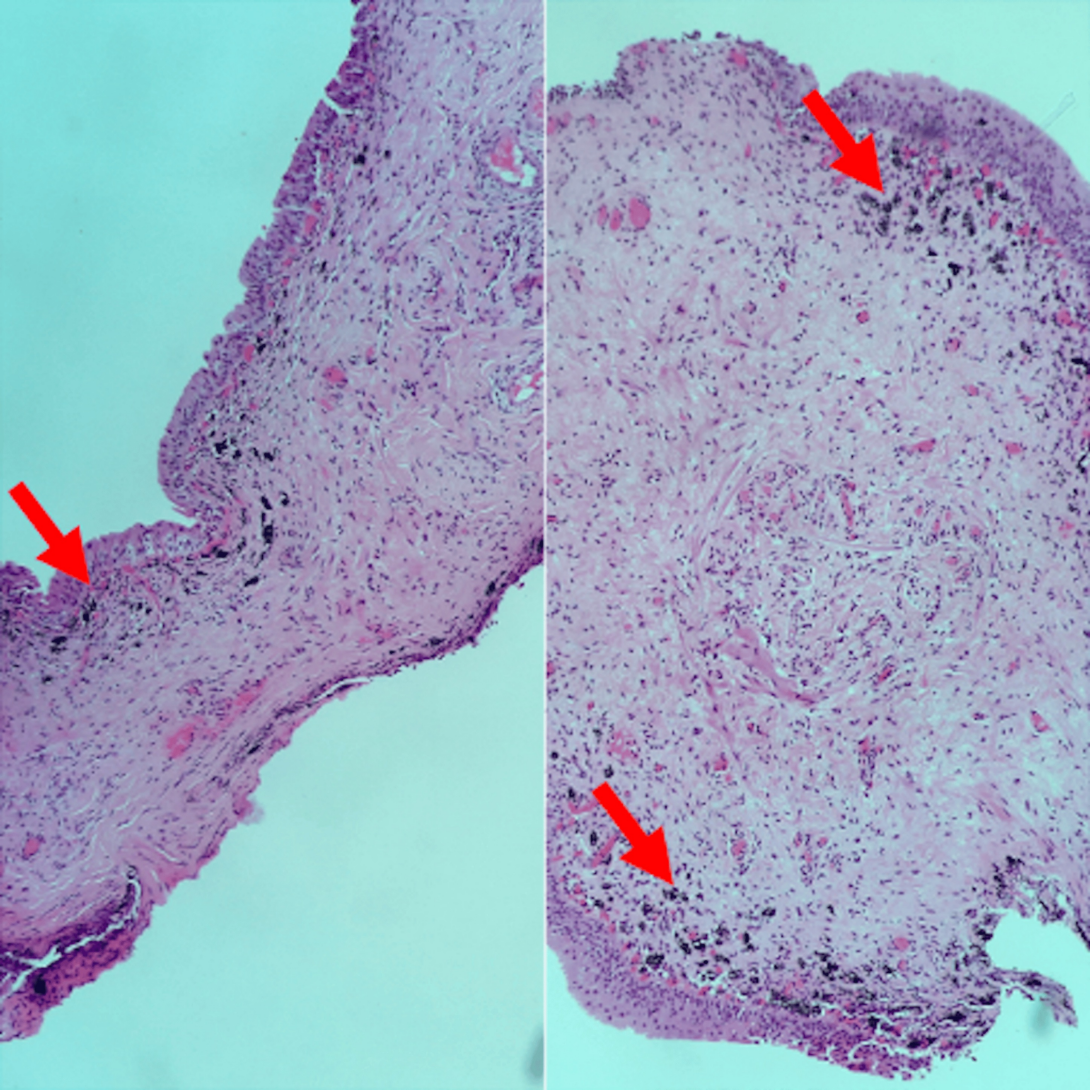
Microscopic histological appearances of the patient’s biopsies Composite image – bladder mucosal fragments, lined by basal flat urothelium showing melanin deposition in the superficial lamina propria (red arrows).

Histology of the bladder lesions (Figure 3) revealed mildly inflamed bladder mucosa with appearances in keeping with melanosis. There were no features of carcinoma in situ, papillary lesions, or invasive neoplasia. 

Follow-up eight months after the cystoscopy, without any further intervention, showed a residual volume of 12 ml with a significant subjective improvement of symptoms. While there is no definitive evidence suggesting a potential for malignant transformation, and in the absence of established guidelines, the patient has been rebooked for a follow-up cystoscopy after one year out of an abundance of caution.

## Discussion

Melanosis, the presence of melanin pigmentation within mucosal surfaces, has been well-characterised in the oral cavity, conjunctiva, and colonic mucosa [7,8]. However, it has been reported much more infrequently in the urogenital tract. A medical literature search was performed by searching for “bladder melanosis” on PubMed, which yielded 33 previous cases with 26 cases of simple melanosis, six of melanosis associated with carcinoma and one of bladder melanoma associated with melanosis. 

Cases are described in Table 1. Unless specified, cystoscopy and histological findings were in keeping with those described below. Patients ranged from five to 86 years old, with an equal distribution of male to female patients. Cases of bladder melanoma, unless associated with melanosis, were excluded. In this article, we aimed to synthesise all currently reported cases. 

**Table 1:** Review of cases CT: computed tomography; UTI: urinary tract infection; GAG: glycosaminoglycan; LUTs: lower urinary tract symptoms; ESWL: extracorporeal shock wave lithotripsy; TCC: transitional cell carcinoma; BCG: bacillus Calmette-Guerin; TURP: transurethral resection of the prostate; TURBT: transurethral resection of bladder tumour

Author	Year	Country	Age, sex	Past medical history	Urological symptoms/presentation	Investigations	Management	Follow-up and outcome
Melanosis								
Alroy et al. [4]	1986	USA	71, male	Hypertension	Bloody urethral discharge	Cystoscopy - multiple, flat and irregularly shaped small foci of dark brown pigmentation in the prostatic urethra and similar clusters of punctate pigmentation throughout the vesical urothelium	Not reported	Not reported
Alroy et al. [4]	1986	USA	72, male	Not reported	Urinary obstruction	None other than cystoscopy and histology	Not reported	Not reported
Alzubaidi et al. [9]	2022	USA	67, male	Uncomplicated nephrolithiasis. 90-pack year smoking history.	Intermittent haematuria	Cytology – negative. Culture – asymptomatic bacteria. CT - bilateral renal calculi, unilateral hydronephrosis.	Percutaneous nephrolithotomy for a stone with brown-black granular pigment deposition in the renal pelvis	Plan to follow up in outpatient clinic
Atieh et al. [10]	2019	USA	71, female	Fibromyalgia. Spinal stenosis. Migraines. Hypersomnia. Sleep apnoea. Recurrent urinary tract infection. Previous cystoscopy and histology showed melanosis.	Back pain and lower urinary tract symptoms.	No investigations other than cystoscopy and histology.	Unknown	Unknown
Cao et al. [5]	2023	USA	82, female	Stage 4 pelvic organ prolapse.	Urge incontinence, voiding dysfunction.	Post-void bladder scan 600 ml.	None reported.	Asymptomatic at one year.
Chong et al. [11]	1999	USA	86, female	Unknown	Urinary incontinence, dysuria.	Urine culture – negative. Cytology – atypical transitional cells with intracytoplasmic brownish-black pigment. Cystoscopy – trabeculated mucosa.	Unknown	Unknown
Duijn et al. [12]	2022	Netherlands	79, male	Cataract. Unilateral adrenalectomy.	Macroscopic haematuria.	Urinalysis – negative. Cytology – candida, melanin. Cystoscopy – trabeculation, diverticula, enlarged median prostatic lobe, mass on the right posterior wall. CT Urogram – normal.	Discharged with intermittent self-catheterisation. Alpha blockers.	Haematuria resolved without further treatment.
Dupey et al. [13]	2022	UK	69, female	Botox for overactive bladder. Recurrent UTIs. Pre-botox cystoscopy normal. Post-botox cystoscopy at 15 months revealed melanosis.	Urgency, urge incontinence, nocturia, voiding difficulty.	Urodynamics – detrusor overactivity.	Commenced on low-dose antibiotics, intermittent self-catheterisation, weekly Intravesical iAluRil.	Two years, Improving macroscopic pigmentation.
Engelhardt et al. [14]	2006	Austria	48, male	None	Frequency, urge incontinence, obstructive voiding symptoms and nocturia. Recurrent UTIs.	Nil significant	Alpha blockers. Intravesical electrical stimulation. Intravesical GAG therapy. 10 years after diagnosis underwent a cystoprostatectomy for ongoing symptoms.	Melanosis with patchy epithelial metaplasia.
Fiore et al. [15]	2007	Italy	72, male	Endoscopic resection of prostate 11 years earlier. Normal cystoscopy at the time.	Dysuria despite multiple courses of antibiotics.	Urine culture – negative.	Unknown	10 years – no recurrence.
Godinho et al. [16]	2016	Portugal	64, male	Ex-smoker. Previous transurethral resection of the prostate with macroscopic haematuria and LUTs.	Ultrasound – bladder wall thickening. Cytology - reactive transitional cells, with lots of histiocytes, containing a brown-blackish pigment, positive for the Masson-Fontana technique	Routine follow-up with annual cystoscopy	Regular follow-up	Two-year follow-up with annual cystoscopy and cytology, with no signs of recurrence of lesions with normal macroscopic urothelium findings.
Gupta et al. [17]	2010	Germany	84, male	Unknown	Painless haematuria	None other than cystoscopy and histology	Unknown	Unknown
Hagmann et al. [18]	2023	Switzerland	55, female	Multiple sclerosis. Recurrent cystitis requiring antibiotics.	Neurogenic bladder. Urge symptoms treated with mirabegron. Insufficient response to treatment.	Ultrasound – normal. Urodynamics - hypo capacitive, hypersensitive, hypo contractile bladder with terminally unstable detrusor with detrusor-sphincter dyscoordination and reduced compliance	Pelvic floor physiotherapy. Trospium chloride. A single course of co-amoxiclav.	Unknown time. Planned for annual cystoscopies. Body mapping by dermatology.
Hogan et al. [19]	2022	Ireland	81, male	Hyperlipidaemia, hypertension, mitral valve regurgitation.	Obstructive symptoms – poor flow, incomplete emptying, nocturia.	Ultrasound – trabeculated bladder, cystic changes. Prostate specific antigen – normal.	Tamsulosin and dutasteride.	Symptoms improved
Jin et al. [20]	2009	USA	77, female	None	Urine incontinence, leakage, urgency. Tolterodine provided no improvement	No investigations other than cystoscopy and histology.	Unknown	Unknown
Lopategui et al. [21]	2020	USA	45, male	Meningoencephalitis	Weak urinary stream, urge incontinence.	Urodynamics – detrusor overactivity, equivocal parameters for bladder outlet obstruction and normal bladder detrusor contractility.	Alpha blockers. Anticholinergics.	Unknown follow-up. Obstructive symptoms improved. Storage and frequency symptoms persisted.
Marti et al. [22]	2004	Spain	75, male	None	Obstructive and irritative symptoms.	Ultrasound - bilateral hydronephrosis. Cystoscopy - trabeculated bladder. Post-void bladder scan - retention.	Alpha blockers.	Two years of regular follow-up. Symptoms improved.
Mera et al. [23]	2018	UK	66, male	Cystoscopy eight years previously, which had demonstrated a bulbar urethral stricture. It was initially managed by intermittent self-dilatation and optical urethrotomy.	Lower urinary tract symptoms, unspecified.	Urinalysis – inconclusive. Cytology – inconclusive.	Unknown	Complete spontaneous resolution of the abnormal discolouration has been demonstrated in the follow-up cystoscopy one year after the initial diagnosis of bladder melanosis.
Patel et al. [24]	2013	Canada	60, male	Non-smoker	Flank pain	Urinalysis – large leukocytes, negative nitrites, moderate blood. CT – Obstructing calculus proximal ureter with hydronephrosis.	Ciprofloxacin six weeks.	Repeat cystoscopy three months – clearing of mucosal pigmentation. Lost to follow-up
Rossen and Petersen [7]	1999	Denmark	44, female	None reported	Recurrent cystitis and difficulty voiding.	Cystourethroscopy – urethral stricture. Biopsies – squamous metaplasia. Electron microscopy - Intra-urothelial cells containing pre-melanosomes and mature melanosomes without intercellular junctions.	Unknown	Unknown
Rossen and Petersen [7]	1999	Denmark	43, male	None reported	Asymptomatic gross haematuria.	None other than cystoscopy and histology.	Unknown	Unknown
Sawalem et al. [25]	2019	UK	69, male	Recurrent UTIs. Bladder diverticulum -> open diverticulectomy, cystoscopy normal. Pyronine’s disease treated with ESWL.	Lower urinary tract symptoms. Treated with Intermittent Self Catheterisation.	Urodynamics – Mild detrusor overactivity. CT abdomen and pelvis – normal. Urine culture – Aerococcus urinae.	Intermittent self-catheterisation	Unknown
Talmon et al. [26]	2010	USA	56, female	None reported	Overactive bladder.	Urinalysis – normal.	The patient underwent implantation of a neurostimulator.	Asymptomatic at six months following the cystoscopy.
Varik et al. [6]	2022	UK	5, male	None	Urinary incontinence and frank haematuria.	Ultrasound – solitary right kidney. Urodynamics – underactive voiding, detrusor overactivity. Following treatment, two years after presentation – pigmented patches on cystoscopy.	Did not respond to urotherapy, desmopressin, tolteridine, solifenacin, mirabegron, or transcutaneous electric nerve stimulation (TENS). Intermittent self-catheterisation. Intravesical botox	Unchanged at 29 months
Willys et al. [27]	2011	Australia	71, female	Rectal prolapse treated with Delorme’s procedure. Fundoplication for gastro-oesophageal reflux disease. Cerebral angioma with six subarachnoid haemorrhage. Hypothyroidism. Epilepsy.	Chronic incontinence and intermittent voiding dysfunction, received trans-obturator mid-urethral sling insertion. Chronic urinary retention with recurrent urinary tract infections.	Cystoscopy - moderate trabeculation and inflammatory eschar at the trigone.	Unknown	Unknown
Wollner et al. [28]	2016	Switzerland	80, female	Hereditary spinal paralysis. Hypertension.	Neurogenic bladder dysfunction. Urge incontinence and nocturia.	Ultrasound - normal. Urodynamics – high residual volume, Detrusor overactivity. Cystoscopy – Trabeculated bladder.	Trospium chloride - No improvement. Catheterisation – No improvement. Fesoterodine therapy and suprapubic cystostomy – symptoms improved.	Symptoms resolved. No follow-up cystoscopies.
Melanosis with carcinoma								
Asad et al. [29]	2023	USA	77, male	Smoker. Hypertension. Type 2 diabetes. Papillary thyroid cancer treated with lobectomy. Previous high-grade TCC – treated with BCG and BCG/interferon for six weeks each.	CT showed bladder wall thickening and trabeculation along the dome and posterior bladder. Urinalysis normal. Bloods normal.	TURP, biopsies showed chronic cystitis with melanosis and invasive papillary urothelial carcinoma.	Unknown	Unknown
Harikrishnan et al. [30]	2012	UK	50, male	Hypertension	Loin pain, haematuria.	CT – duplex kidney, renal calculi, thickened renal pelvis. CT with contrast – tumour in the renal pelvis and distal ureter. Cystoscopy – pigmentation in keeping with melanosis, ureteric TCC	Nephrouretectomy	Unknown
Mateescu et al. [31]	2023	UK	58, female	Hypertension, hypercholesterolaemia, osteoarthritis. Non-smoker. 40 units of alcohol per week.	Recurrent UTIs	Cystoscopy - Reticular black discolouration area on the right side, anterior wall, and dome of the bladder. pTa G1 TCC of the bladder.	Trimethoprim and intra-vesical iAluRul.	Follow up at six months – complete resolution. Remains recurrence-free after two years.
Patel et al. [24]	2013	Canada	69, female	None	Gross haematuria	Cystoscopy – black-coloured tumour involving the trigone and extending toward the right lateral wall. Both ureteric orifices were obscured.	Conservative management.	Passed away two months following the diagnosis
Sanborn et al. [3]	2009	USA	63, female	Recurrent urinary tract infections. Initial cystoscopy – melanosis.	Haematuria	Repeat cystoscopy at one year - mucosal high-grade invasive papillary TCC.	Intravesical BCG. Follow-up cystoscopy – resolved melanosis.	Ongoing surveillance cystoscopy every three months, provide urine for fluorescent in situ hybridization analysis, and undergo studies of her upper genitourinary tracts.
Yau et al. [32]	2017	USA	50, female	Hypertension. Smoker.	Visible haematuria. Urinary obstruction.	Urine culture. Negative. Cystoscopy – obstructive effect and right hydroureter.	Repeat TURBT - muscle-invasive urothelial carcinoma with squamous features. A right nephrostomy tube was placed to alleviate obstruction and she began neoadjuvant chemotherapy with planned cystectomy and urinary diversion.	Not reported
Melanosis with melanoma								
Kerley et al. [33]	1991	USA	80, female	Unknown	Vulvar melanoma and bladder melanosis, underwent vulvectomy. Presented three years later with bladder melanosis.	Cystoscopy – bladder melanosis. Unclear whether melanocytes were present.	Radical surgery	Passed away 18 months after radical surgery

The aetiology and pathogenesis of bladder melanosis is uncertain. Urological mucosa does not contain melanocytes, and the presence of these has been postulated by some authors to be secondary to migration of these cells from the neural crest or from aberrant migration of urothelial stem cells during development [7,34,35]. Differentials include blue nevus or primary or secondary melanoma. Case reports have reported instances of melanosis in conjunction with urothelial carcinoma and the subsequent development of melanoma in one case, although it is unclear whether there is a causal relationship. 

Histologically, in melanosis, dark brown to black pigments are found in the urothelial cells, with or without pigment-laden macrophages in the lamina propria. Fontana-Masson staining is positive, with negative periodic acid-Schiff (PAS) and iron stains. The pigment disappears upon melanin bleaching. Atieh et al. argued that melanosis does not necessarily indicate the deposition of melanocytes and may instead represent the accumulation of other deposits such as lipofuscin as is the case in melanosis coli [10]. Lipofuscin is diagnosed histologically using a positive PAS, Sudan black B or Ziehl-Neelson acid-fast stains. It is bleach-resistant. Haemosiderin is another differential and is an intracellular iron produced from the digestion of hematin. Staining would be positive when staining for iron [5]. Melanoma can be identified by the presence of positive S-100 and human melanoma black (HMB)-45 tests [5].

Presenting complaints of those diagnosed with simple melanosis were of urge incontinence, lower urinary tract symptoms, dysuria, obstructive symptoms, nocturia, and haematuria. Cystoscopy reveals brown to black mucosal pigmentation, which can be flat or have a small punctate appearance in some or all areas of the bladder mucosa [3]. When performed, urodynamics commonly showed detrusor over-activity and high residual volumes.

Management was symptom-orientated and consisted in the majority of cases of alpha-blockers, anticholinergics, anti-muscarinics, and occasional short courses of antibiotics. Occasionally, pelvic floor physiotherapy, intermittent self-catheterisation, botox, or other intravesical instillations were utilised. In the majority of cases where cases were followed up, symptoms improved. In some cases, the macroscopic melanosis improved or even resolved completely. 

There exist six reports of melanosis either preceding or in conjunction with transitional cell carcinoma (TCC) of the urogenital tract, although no causal relationship has been proven. Ages at presentation ranged from 50 to 77. Three were referred for haematuria, two for recurrent urinary tract infections (UTIs), and one had a background of previous TCC. In one case, melanosis was discovered on follow-up cystoscopy for monitoring of a previous TCC [29]. In further cases, it was discovered simultaneously following a biopsy [24,30-32].

In another case, melanosis preceded the development of high-grade TCC by a year [3]. Sanborn reported a case of melanosis diagnosed in a 63-year-old female with recurrent UTIs. Cystoscopy and histopathological analysis revealed melanosis. On repeat cystoscopy a year later, due to haematuria, a mucosal TCC tumour was noted with staging pT1N0M0. They were treated with intravesical BCG. At post-therapy cystoscopy, the melanosis was noted to have resolved [3].

Smoking was identified as a relevant factor in the past medical history of four patients, two of which had simple melanosis (Alzubaidi et al. [9] and Godinho et al. [16]) and two of which had melanosis in association with carcinoma (Asad et al. [29] and Yau et al. [32]). The patient presented by Alzubaidi et al. had a 90-pack-year history, while Godhino et al. presented the case of a patient who was a "former smoker" of unspecified quantities. Asad et al. presented the case of a "current" smoker of unspecified quantities, and Yau et al. presented the case of a 20-year history of intermittent smoking. Smoking has been identified as an important risk factor for the development of TCC. Polesel et al. conducted a case-control study of 531 cases of TCC matched with 524 cancer-free patients and discovered that the risk of developing TCC was three times higher in former smokers, and six times higher in current smokers [36].

Our patient, a fit and well 51-year-old male, was referred following two episodes of visible haematuria on a background of long-term urinary frequency and nocturia. Cystoscopy and biopsy revealed simple melanosis. At follow-up, their symptoms and uroflow rate had improved despite no medical intervention. They will be followed up with regular routine reviews and cystoscopies to monitor their symptoms and ensure no malignant transformation. 

Because of the low number of cases reported and the unavailability of long-term follow-up, the longest being 10 years [14], the potential long-term implications are unknown. Many authors have attributed melanosis as a benign lesion, with some reports of associations with malignancy. Regular follow-up may be prudent to ensure no subsequent development of malignancy. 

## Conclusions

We have described a case of bladder melanosis in a fit and well 51-year-old who presented with visible haematuria on a background of long-term urinary frequency and nocturia. Bladder melanosis is a rare entity of which there have only been 33 reported cases. It is considered a benign disease and most cases resolve or become asymptomatic, sometimes without treatment. Associations with transitional cell carcinoma and melanoma have been reported. As no guidelines currently exist, careful and prolonged follow-up may be warranted to ensure no malignant transformation. 
